# First Time Seizure in the Setting of a Congenital Heart Abnormality and MCA Mycotic Aneurysms

**DOI:** 10.3390/jcm5040046

**Published:** 2016-04-12

**Authors:** Michael R. Minckler, Merlin Curry, Jarrod M. Mosier

**Affiliations:** The University of Arizona, Department of Emergency Medicine, Tucson, AZ 85721, USA; mcurry@aemrc.arizona.edu (M.C.); jmosier@aemrc.arizona.edu (J.M.M.)

## 1. Clinical Presentation

A 37 year-old man presented to the Emergency Department (ED) with new onset seizure and fall from standing. He had a witnessed 20-second generalized tonic–clonic seizure and bit his tongue. He was post-ictal for approximately five minutes. On arrival at the ED, he was awake and alert with a Glascow Coma Scale (GCS) of 15. He reported a lingering headache for 3–4 days prior to this event similar to prior migraines except for the prolonged time course.

**Figure 1:**
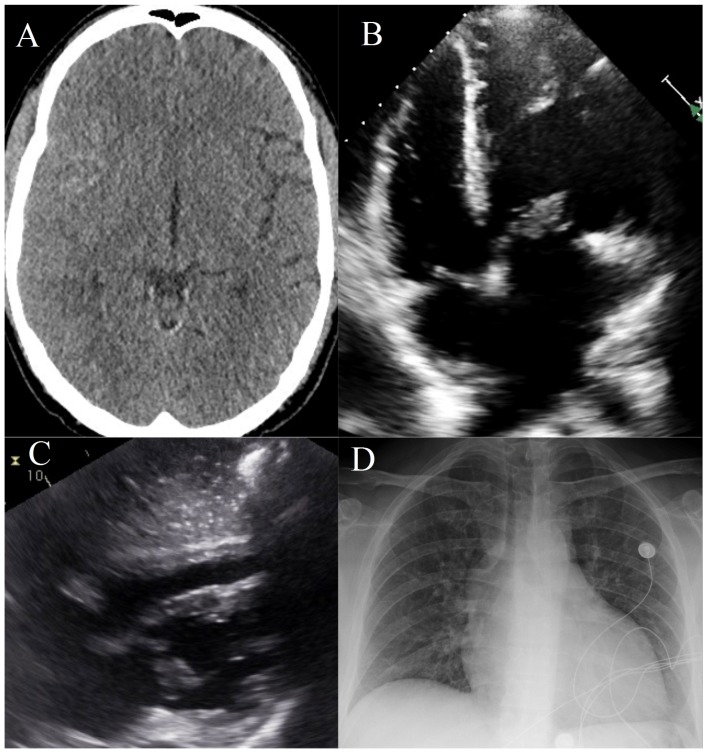
A composite image of radiographic and ultrasonographic findings from this case. (**A**) A coronal view of an obliterated right Sylvan Fissure due to hemorrhage from a ruptured mycotic aneurysm on head CT scan; (**B**) A tricuspid valve with an infective vegetation on echocardiogram; (**C**) An echocardiogram showing transposition of the great vessels with congenital correction resulting in atrio-ventricular discordance; (**D**) A chest X-ray showing the patient’s abnormal cardiac silhouette.

The patient was found to have an intracranial bleed obscuring his right Sylvan Fissure (Figure 1A). He was seen to have an enlarged cardiac silhouette on chest x-ray (Figure 1D). Cardiac echo revealed an L-transposition of the great arteries with morphologic right ventricle on the left side and morphologic left ventricle on the right. His congenital abnormality was congenitally corrected, and he had atrio-ventricular discordance (Figure 1C). We can see a vegetation, from infective endocarditis (IE) (Figure 1B), on his tricuspid valve that is part of his systemic vascular circuit. This vegetation seeded multiple small right MCA mycotic aneurysms that were rupturing and causing his symptoms.

**Figure 2:**
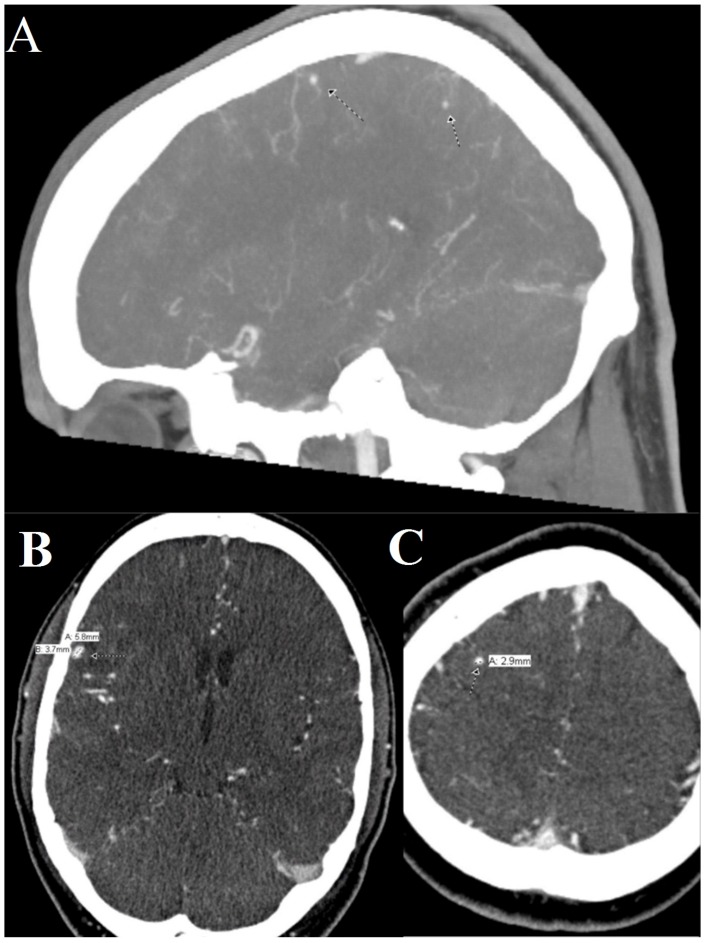
A composite image of the patient’s mycotic aneurysms found on computed tomography angiogram. (**A**) A sagittal view of multiple mycotic aneurysms in the right middle cerebral artery distribution; (**B**) A coronal cross section of a 6 mm by 4 mm mycotic aneuryms in the right middle cerebral artery distribution; (**C**) A coronal cross section of a 2.9 mm mycotic aneurysm in the right middle cerebral artery distribution.

During his hospital stay he was started on broad-spectrum antibiotics and his blood cultures grew *Streptococcus mutans*, making this the organism responsible for his endocarditis and aneurysms. The sagittal views of the mycotic aneurysms are shown in Figure 2A. The larger one, 6 mm by 4 mm (Figure 2B) was responsible for his seizure as it was rupturing; the other (Figure 2C), was 3 mm and unruptured. For this he underwent right craniotomy with aneurysm clipping, performed by neurosurgery. His follow up endovascular interventional radiographic study showed resolution of the aneurysms.

## 2. Discussion

Congenital heart disease is present in our population at a rate of approximately 2.5/1000–3/1000 live births [1]. Among the rarest forms of congenital heart disease are congenitally corrected transpositions of the great arteries—levo-transposition of the great vessels (LTGV)—representing less than one percent of all congenital heart disease.

Patients with congenital heart disease carry an elevated life-long risk of suffering from infective endocarditis (IE). The incidence of IE in patients with congenital heart disease is increasing, likely due to advanced surgical techniques that have allowed many children with congenital heart malformations to live into adulthood [2]. Increased risk of IE is still present even after surgical correction of congenital malformations.

Approximately 10% of patients with endocarditis will develop neurovascular mycotic aneurysms. These mycotic aneurysms tend to be small, only a few millimeters, and prove fatal in 80% of cases when rupture occurs [3]. In general, the distal branches of the pial vessels, especially the MCA, are the most likely sites for seeding. While it has been reported that some mycotic aneurysms may resolve with antibiotic therapy alone, given the high fatality rate of a ruptured aneurysm and the difficulty of estimating disease course, neurosurgical evaluation and intervention are indicated for these patients [4].
